# Comparison between Conventional and Real-Time PCR Assays for Diagnosis of Visceral Leishmaniasis

**DOI:** 10.1155/2014/639310

**Published:** 2014-02-06

**Authors:** Mariana R. Pereira, Fabiana Rocha-Silva, Cidiane Graciele-Melo, Camila R. Lafuente, Telcia Magalhães, Rachel B. Caligiorne

**Affiliations:** Núcleo de Pós-Graduação e Pesquisa Hospital Santa Casa de Belo Horizonte, Rua Domingos Vieira 590, 30150240 Belo Horizonte, MG, Brazil

## Abstract

The diagnosis of visceral leishmaniasis (VL) is a challenging issue and several studies worldwide have evaluated the different tools to reach a diagnostic solution. The polymerase chain reaction (PCR) has proven to be effective in detecting the genome of *Leishmania* species in different biological samples. In this study, we compared the conventional PCR and real-time PCR using the Sybr Green system and their application in molecular diagnosis of visceral leishmaniasis in peripheral blood as a biological sample. The genus-specific conserved region of kinetoplast DNA (kDNA) was the target of amplification. We studied 30 samples from patients with suspect of visceral leishmaniasis who were treated by the Medical Clinic of Santa Casa de Belo Horizonte Hospital, Brazil. Among the samples studied, 19 had a confirmed diagnosis for VL by serology and/or by clinical findings. Among these 19 samples, 63% (*n* = 12) presented positive results for serology and 79% (*n* = 15) positive results in both PCR methodologies. This fact suggests that the PCR technique can assist in the diagnosis of visceral leishmaniasis in patients who do not have detectable antibodies by serology but can present the genome of the parasite circulating in whole blood. Also, it was possible to observe that there was conformity between the results of the techniques of cPCR and qPCR using the Sybr Green system in 100% of samples analyzed. These data suggest that both PCR techniques were equally effective for detection of the genome of the parasite in the patient's blood.

## 1. Introduction

Visceral leishmaniasis (VL) and cutaneous leishmaniasis (CL) are defined as a zoonosis caused by parasites of the genus *Leishmania* sp. This disease is caused by *Leishmania donovani *in Asia and Africa and by  *Leishmania infantum/chagasi *in the Mediterranean, China, North Africa, and Latin America [1–4]. The different forms of leishmaniasis occur endemically in 90 countries spread over five continents: Africa, Asia, Europe, North, and South America [3–5]. It is estimated that about 12 million people worldwide are infected with some form of leishmaniasis [6].

The diagnosis of visceral leishmaniasis is based on clinical, epidemiological, and laboratory approaches [7–9]. Several authors consider the detection of parasite DNA in biological samples as alternative for leishmaniasis diagnosis [10–13]. The standardization of molecular biology such as amplification of species-specific genomic regions by the polymerase chain reaction (PCR) is of great importance, since that will support the diagnosis of the disease and the identification of *Leishmania *species, in cases that the serological and parasitological tests do not elucidate the diagnosis. This technique has the advantage of replicating the genome of the agent from the minimum quantity of circulating DNA in biological samples [6, 14, 15].

Because of its abundance, specificity, and repetitive nature, the kinetoplast DNA (kDNA) has often been target of detection of *Leishmania *species [16]. According to some studies, the use of kDNA target amplification has shown high specificity and effectiveness [17]. El-Beshbishy et al. (2013) [18] compared the PCR amplification using as targets primers that anneal in the regions of ribosomal DNA (rDNA) of the parasite and also that anneal in the kDNA. The results of this study demonstrate that kDNA-PCR had a sensitivity of 90.7%, whereas for the rDNA-PCR, the sensitivity was 70.1%.

A major concern for public health services with leishmaniasis is the necessity for rapid and accurate diagnosis of this disease with low cost. The aim of this study was to evaluate the application of conventional PCR and real-time PCR using the kDNA as target of amplification, in the diagnosis of visceral leishmaniasis, an affordable cost to the patient treated by the public health system.

## 2. Methodology

### 2.1. Biological Samples

Thirty blood samples were collected from patients with suspect of visceral leishmaniasis, treated at the Santa Casa de Belo Horizonte Hospital, Brazil, from September, 2012, to April, 2013. The project was approved by the Ethics Committee (CEP) of the Santa Casa de Belo Horizonte Hospital, with the protocol number 021/2010.

Among the thirty cases analyzed in our study, 12 were diagnosed with VL confirmed by clinical findings and the indirect fluorescence antibody test (IFAT). Seven cases were clinically suspected for VL, devoid of serological positive tests. In these cases, the patients received treatment for the disease and showed good clinical improvement. The remaining cases (*n* = 11) had a differential diagnosis for VL, with other diseases such as atrophic gastritis and anemia of various etiologies.

### 2.2. DNA Extraction from Whole Blood

The DNA was extracted using the Invitrogen kit (USA). After being extracted and purified, the DNA was assayed by spectrophotometry using Nanovue Plus (GE Healthcare Life Sciences, Sweden). Despite the concentration of the DNA purified, all samples were diluted 10 times in sterile ultrapure water, before being used in the PCR reactions.

### 2.3. Conventional PCR (cPCR)

The DNA extracted from whole blood was subjected to PCR assay using primers directed to the conserved region of *Leishmania *genus minicircle kDNA (mkDNA): the sense primer 150-GGGKAGGGGCGTTCTSCGAA and anti-sense primer 152-SSSWCTATWTTACACCAACCCC [19, 20].

The negative and positive controls were included in all PCR assay performed. The DNA extracted from promastigotes of a sample-reference *Leishmania (Leishmania) infantum* MHOM/BR/2002/LPC-RPV was used as positive control. Tubes containing only sterile ultrapure water instead of the DNA samples were used as negative control in the PCR assays.

For all PCR assays were used specific primers for the constitutive **β*-globin* gene, as a quality control of the reaction. For each PCR reaction, were used the following reagents: 2 mM MgCl_2_, 200 *μ*M dNTPs, 0.6 *μ*M of each primer (Sigma, USA), 1 UI *Taq DNA polymerase *and specific buffer (Invitrogen, USA), and 20 ng DNA template. The program was used as follows: step one: 10 minutes 94°C; step two: 30 seconds 60°C; step three: 30 seconds 72°C; step four: 30 seconds 94°C; go to step two for 42 times; and, finally, 10 minutes 72°C. The final product of amplification was analyzed on a 7% polyacrylamide gel and stained with 0.2% silver nitrate.

### 2.4. PCR Real Time (qPCR)

For the standardization of real-time PCR was employed the Sybr Green system (Ludwig, Brazil). For comparison between the two techniques, the same pair of primers described for cPCR was applied. The universal cycling conditions were used for amplification (95°C for 10 minutes followed by 40 cycles of 95°C for 15 seconds and 60°C for 1 minute) and performed quantitative analysis of the presence or absence of the pathogen, based on the presence of amplification. In this work was performed dissociation curve on all boards' amplification.

All samples were analyzed in duplicate. For all PCR reactions were used specific primers for the constitutive *β-globin* gene, as a quality control of the reaction. The negative and positive controls were included in all PCR reactions performed. The DNA extracted from promastigotes of a sample-reference *Leishmania (Leishmania) infantum* MHOM/BR/2002/LPC-RPV was used as positive control. Tubes containing only sterile ultrapure water instead of the DNA samples were used as negative control in the PCR reactions.

## 3. Results and Discussion

For purposes of comparative analysis of diagnostic techniques, we considered the 19 samples of patients who had a confirmed diagnosis by serology and/or by clinical findings. Thus, among these 19 samples, 63% (*n* = 12) presented positive results for indirect fluorescence antibody test (IFAT) and 79% (*n* = 15) positive results in both PCR methodologies. The remaining samples (*n* = 11) which presented a differential diagnosis for VL showed PCR negative results, allowing the expected finding.

The leishmaniasis is highly related to the immunosuppressive diseases such as AIDS, leukemia, among others [3–5]. In many cases the PCR may aid in the diagnosis of VL in which the patient has no detectable amounts of antibodies by serological techniques but can present the genome of the parasite circulating in whole blood. Therefore, molecular biology has been presented as an important tool in the detection of infectious and parasitic diseases, in immunosuppressed patients.

According to the results obtained, there was conformity between the results of the techniques of cPCR and qPCR using the Sybr Green system in 100% of samples analyzed. These data suggest that both PCR techniques were equally effective for detection of the genome of the parasite in the patient's blood, requiring critical adjustments in accordance with the conditions intralaboratory, so that the efficiency and sensitivity are maintained. It is important to report that the DNA purified from the blood samples presented amplification when it was tested in full concentration or, sometimes, when it was 10 times diluted in sterile ultrapure water (data not show). Thus, this study suggests that all DNA of the blood samples should be tested in full concentrations and 10 times diluted in sterile ultrapure water, despite DNA quantity. These data support some publications that demonstrate the need for detailed standardization for improving performance of PCR in biological samples [16, 21–23].

The use of the kDNA as amplification target has demonstrated favorable results, demonstrated by many studies [15, 18–20, 24–26]. However, the great genetic diversity among species of *Leishmania* genus hampers the development of a diagnostic method that can encompass all forms of leishmaniasis and detect overall species agent of the disease [27]. Thus, at present, there is no standardization described for probes that anneal in genomes of each species of *Leishmania*, which makes it impossible to have a standardized system by TaqMan qPCR for detecting large-scale species of the *Leishmania* genus [16, 21]. Facing that, the technique of qPCR by means of the Sybr Green system, using the target kDNA provides the diagnosis of leishmaniasis enable to detect all species of the genus in the same time.

Some authors have already demonstrated the right application of qPCR for the diagnosis of leishmaniasis, using the Sybr Green system [16, 28, 29]. It is noteworthy that the Syber Green system has a lower cost, since the use of probes or multiple probes for accomplishing all different species of the genus implies a high cost of the test and therefore will be less feasible to be applied to the system of public health.

In our experiments we observed that the results of cPCR and qPCR using the Sybr Green system were similar, demonstrating that both techniques have the same effectiveness to assist in the leishmaniasis diagnosis, when using peripheral blood as a biological sample. Therefore, the choice of method should be evaluated in accordance with the reality of service and technical resources available, as already shown in previous studies [15, 29].
